# Genetic diversity of *Toxoplasma gondii* isolates obtained from free-living wild birds rescued in Southeastern Brazil

**DOI:** 10.1016/j.ijppaw.2018.11.001

**Published:** 2018-11-10

**Authors:** W.M.F. Rêgo, J.G.L. Costa, R.C.A. Baraviera, L.V. Pinto, G.L. Bessa, R.E.N. Lopes, J.A.G. Silveira, R.W.A. Vitor

**Affiliations:** Departamento de Parasitologia, Instituto de Ciências Biológicas, Universidade Federal de Minas Gerais (ICB-UFMG), CEP, 31270-901, Belo Horizonte, MG, Brazil

**Keywords:** *Toxoplasma gondii*, Genotypes, Wild birds, ROP18, ROP5, PCR-RFLP

## Abstract

Recent studies carried out in Brazil have shown that strains from the same *Toxoplasma gondii* genotype can infect humans, domestic animals (dogs and cats) and animals slaughtered for human consumption (pigs, sheep, goats, and chickens), suggesting a common infection route. However, little is known about the importance of free-living wild birds within this epidemiological context. The objective of this work was to isolate, genotype, and evaluate the virulence for mice of new isolates of *T. gondii* obtained from free-living wild birds from the state of Minas Gerais, Southeastern Brazil. From August 2016 to June 2017, *T. gondii* was isolated from the hearts and brains collected from 6 out of 45 free-living wild birds, namely, a roadside hawk (*Rupornis magnirostris*), a campo flicker (*Colaptes campestris*), a southern caracara (*Caracara plancus*) and a tropical screech-owl (*Megascops choliba*), all rescued in Belo Horizonte. One isolate was obtained from a toco toucan (*Ramphastos toco*), rescued in Cristiano Otoni, and another was obtained from southern caracara, rescued in Santa Luzia. Five different genotypes were identified by PCR-RFLP. A unique genotype was shared in two different isolates obtained from a southern caracara and a toco toucan. This genotype has never been previously described in any other host or place. Three isolates were classified as of intermediary virulence and three isolates as avirulent for mice. The combined analysis of alleles ROP18/ROP5 (a serine/threonine kinase, and a polymorphic pseudokinase, respectively) was effective in determining the virulence of five of all the isolates with the exception of that from *R. magnirostris*. Atypical isolates of *T. gondii* obtained from free-living wild birds rescued in the state of Minas Gerais share the same genotypes of strains that infect humans, domestic animals, and animals slaughtered for human consumption.

## Highlights

• Forty-five bird samples were bio assayed and six *T. gondii* strains were isolated.
• Five different genotypes were identified.
• Wild birds share the same genotypes that infect humans.
• None of the six isolates was 100% lethal to mice.
• ROP18/ROP5 was not effective in determining the virulence of strain TgWildBrMG1.

## Graphical abstract

**Figure:**
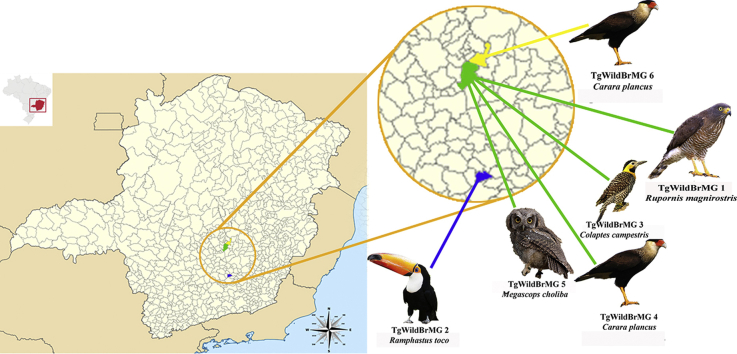

## 1 Introduction

*Toxoplasma gondii* is an apicomplexan protozoan capable of causing neurological disturbances in the fetus, newborns, and in immunodeficient patients, mainly those with acquired immunodeficiency syndrome (AIDS). Serological surveys in Brazil have reported infections in pregnant women ranging from 50 to 80% (Dubey et al., 2012). Congenital ocular toxoplasmosis is more severe in Brazil compared to Europe, probably due to infection with more virulent genotypes of the parasite predominating in Brazil (Gilbert et al., 2008).

Studies have been conducted worldwide, and, especially, in Brazil, to isolate and genotype *T. gondii* from tissues of various species of domestic animals (Dubey et al., 2002; Brandão et al., 2006; Cavalcante et al., 2007; Pena et al., 2008; Macedo et al., 2012; Rêgo et al., 2017). Bioassay to obtain new isolates of *T. gondii* is a current methodology, frequently used in the study of toxoplasmosis (Murata et al., 2018). This method is sensitive and allows obtaining viable parasites which can be used in subsequent biological studies. However, such studies using free-living wild birds are still scarce (Yai et al., 2008, 2009; Vitaliano et al., 2014). Little is known about the isolates of *T. gondii* that infect free-living wild animals. This lack of information has a direct impact on understanding the biological and genotypic characteristics of *T. gondii* isolates present in the wild environment.

*Toxoplasma gondii* has been isolated from humans, domestic animals, and animals slaughtered for human consumption in the state of Minas Gerais. Genotyping these isolates has revealed the existence of *T. gondii* strains of the same genotype in humans and domestic animals in several places in Minas Gerais (Carneiro et al., 2013; Silva et al., 2014). However, little is known about the importance of free-living wild animals within this epidemiological context. Further, research on the epidemiology, prevalence, distribution and virulence of *T. gondii* in free-living wild animals, as well as genetic characterization to determine if common genotypes occur among wildlife and humans and domestic animals, is needed.

Thus, further studies must be carried out on the epidemiology, prevalence, distribution, virulence and genotyping of isolates of *T. gondii* obtained from free-living wild animals. It is also necessary to identify whether these genotypes are exclusive or more common in the wild fauna compared to the isolates previously genotyped and obtained from humans and domestic animals (Carneiro et al., 2013; Silva et al., 2014; Vitaliano et al., 2014).

Epidemiological evidence suggests a potential association between virulence in mice and disease severity in human toxoplasmosis (Saraf et al., 2017). An outbreak of acute toxoplasmosis in humans caused by a virulent atypical strain was reported in Suriname (Amazon rainforest) near the French Guiana border. Severe cases of toxoplasmosis with ocular and multivisceral involvement were verified in immunocompetent individuals, including deaths. The source of infection from this outbreak could not be defined but the inhabitants were exposed to the following risk factors: contact with soil, vegetation and water in the Amazon forest, presence of domestic and wild cats around the village, and hunting and consumption of wild animals (Demar et al., 2007).

Thus, the objective of this work was to isolate, genotype, and evaluate the virulence of new isolates of *T. gondii* obtained from free-living wild birds from the state of Minas Gerais, Brazil.

## 2 Material and methods

### 2.1 Ethical statements

Collection, transport, and manipulation of free-living wild birds were authorized by the Biodiversity Authorization and Information System of the Federal Government, Brazil – SISBIO (n°. 52653–1) and by the Forest State Institute – IEF (n°. 018) of the state of Minas Gerais, Brazil.

Mice were provided by the Experimental Animal Center of the Universidade Federal de Minas Gerais (UFMG, Brazil) and were maintained at the animal facility for infected animals of the Department of Parasitology (UFMG, Brazil). Animal care and experimental procedures were carried out in strict accordance with the recommendations of the Animal Control National Council (CONCEA) - Brazil. The protocol conducted in this study was approved by the Ethics Committee in Animal Experimentation (CETEA) of the Universidade Federal de Minas Gerais, Brazil (Protocol CEUA 67/2016). Mice were fed water and food ad libitum and maintained under 12 h light/12 h dark light cycles. A specialized technical assistant monitored animal health and behavior twice a day for 45 days postinfection (d.p.i.). All efforts were made to minimize animal suffering during the course of this study. Animals were euthanized by an intraperitoneal (i.p.) overdose of ketamine and xylazine, followed by cervical dislocation. To determine “survival” reliably and humanely, an artificial endpoint was used, defined by the inability of a mouse to raise itself in 10 s after being placed on its side (Scott et al., 2008).

### 2.2 Origin, sampling, and collection of heart and brain fragments from wild birds to obtain new isolates of T. gondii

From August 2016 to June 2017, the organs (heart and brain) of 45 free-living wild birds (Table 1) from the state of Minas Gerais, Southeastern Brazil, were collected and bio assayed with no previous knowledge on the animal serological status. They were collected from rescued birds after death at the Wild Animals Screening Center (CETAS), which is linked to the Forest State Institute (IEF) and to the Brazilian Institute of Environment and Renewable Natural Resources (IBAMA), Belo Horizonte, Minas Gerais, Brazil.

**Table 1:** Identification by scientific name and origin of free-living wild birds used for the isolation of *Toxoplasma gondii* by heart and brain bioassay in Swiss mice.

Scientific name	City of rescued – Minas Gerais state	Total
*Caracara plancus*	Belo Horizonte, Betim and Santa Luzia	07
*Rupornis magnirostris*	Sete Lagoas, Belo Horizonte, Juatuba and Vespasiano	07
*Asio clamator*	Belo Horizonte, Nova Lima, Sabará and Sarzedo	04
*Megascops choliba*	Belo Horizonte	04
*Athene cunicularia*	Belo Horizonte and Betim	03
*Ramphastos toco*	Ribeirão das Neves, Belo Horizonte and Cristiano Otoni	03
*Coragyps atratus*	Caeté and Betim	02
*Falco sparverius*	Nova Lima and Belo Horizonte	02
*Glaucidium brasilianum*	Belo Horizonte	02
*Milvago chimachima*	Betim and Contagem	02
*Amazona aestiva*	Belo Horizonte	01
*Ardea alba*	Belo Horizonte	01
*Asio stygius*	Ibirité	01
*Aramides saracura*	Belo Horizonte	01
*Cariana cristata*	Itabirito	01
*Colaptes campestris*	Belo Horizonte	01
*Geranoaetus albicaudatus*	Belo Horizonte	01
*Patagioenas speciosa*	Betim	01
*Pionus maximiliani*	Belo Horizonte	01
Total		45

The heart and brain were identified, stored in Falcon tubes, and transported under refrigeration to the Toxoplasmosis Laboratory of the Biological Sciences Institute - Universidade Federal de Minas Gerais (ICB/UFMG).

### 2.3 Bioassay

Heart and brain fragments obtained from wild birds were cut into small pieces and individually digested with pepsin at 37 °C for 60 min (Dubey et al., 2002). After neutralizing the pepsin and filtering, the digested tissues were suspended in PBS (pH 7.2) containing 20 μL of penicillin (1.000 units) and streptomycin (100 μg/mL), for 60 min, and 1 mL was inoculated subcutaneously in mice (bioassay). A total of 180 Swiss female mice, 4 for each bird, were used. Two mice (Dubey et al., 2014) were inoculated only with material obtained from the heart and two only with material obtained from the brain of each wild bird. Mice that died before 45 days d.p.i. were examined for the presence of tachyzoites in the peritoneum and lung or cysts in the brain. At the forty-fifth d.p.i., each surviving mouse was bled by lancing the tail, and blood was collected onto filter paper for ELISA tests (Brandão et al., 2009). All surviving mice were euthanized. The brains of ELISA-seropositive mice were removed and macerated, and 1.0 mL of PBS (pH 7.2) was added. Brain suspension was used to determine the presence of tissue cysts under light microscopy. *T. gondii* tachyzoites from each new isolate were obtained from the peritoneal cavities of three Swiss mice (in a total of 18 mice) i.p. Inoculated with 300–500 brain cysts, as previously described (Carneiro et al., 2013), for DNA extraction (PCR-RFLP analysis) and assessment of virulence in mice.

### 2.4 Serological test

An ELISA was used to detect anti-*T. gondii* IgG antibodies in surviving Swiss mice inoculated with wild bird samples to isolate the parasite (bioassay), as well as in surviving BALB/c mice used in virulence tests of the new isolates, as described by Brandão et al. (2009).

### 2.5 DNA extraction, PCR-RFLP and data analysis

Extraction of DNA from tachyzoites was performed using the commercial genomic DNA purification kit *Wizard* (Promega^®^), according to the manufacturer's recommendation. DNA samples were rehydrated with 50 μl of ultra-pure water and stored at 4 °C.

New isolates of *T. gondii* were genotyped by PCR-RFLP using 12 markers: SAG1, 5‘+3’ SAG2, alt*.* SAG2, SAG3, CS3, BTUB, GRA6, c22-8, c29–2, L358, PK1, and Apico (Su et al., 2010). The profile of the alleles was compared with the reference clonal strains RH (type I), PTG (type II) and VEG (type III) (Pena et al., 2008; Su et al., 2010). *T. gondii* isolates were genotyped by PCR-RFLP using only the internal primers specific to each marker, as described by (Pena et al., 2008; Su et al., 2010; Carneiro et al., 2013).

The genes expressing the virulence proteins GRA15, ROP5, ROP16, ROP17, and ROP18 of the *T. gondii* isolates were genotyped by PCR-RFLP using only the internal primers specific to each marker, and the profile of the alleles was compared to the reference strains RH, ME49, VEG, and MAS (Dubey et al., 2014; Shwab et al., 2016).

The PCR products for genotyping and genes expressing virulence proteins were digested with specific restriction endonucleases (Su et al., 2010; Shwab et al., 2016) and visualized in 6% silver nitrate-stained polyacrylamide gel (Carneiro et al., 2013).

The genotyped strain profile was compared to databank strains on http://www.toxodb.org and to results published on other isolates of *T. gondii* obtained in Brazil, but genotypes were not deposited in the databank.

### 2.6 Virulence of new isolates of T. gondii in mice

The virulence of the new isolates of *T. gondii* was determined according to Ferreira et al. (2001) in a total of 180 BALB/c female mice. Five mice were i.p. Inoculated with 1, 10, 100 and 1000 tachyzoites of each isolate in 0.2 mL of PBS (pH 7.2). Five animals inoculated with 0.2 mL of PBS (pH 7.2) were maintained as negative controls. For comparison, five mice were inoculated with tachyzoites of RH (lethal) and five with ME49 (nonlethal) strains, as references. Mice mortality was observed over a 30-day period. The survivors were tested by ELISA. Mice that were inoculated but not seroconverted according to ELISA were excluded from the experiment. All ELISA-positive surviving mice were euthanized to look for tissue cysts in the brain. Isolates killing 100% of the infected mice were classified as virulent. Isolates with LD_100_ greater than 1000 tachyzoites were classified as avirulent, and those with an intermediate pattern between the two extremes were classified as having intermediate virulence (Howe and Sibley, 1995).

## 3 Results

Of the 45 free-living wild birds evaluated (Table 1), six were positive by bioassay. Six isolates of *T. gondii* were obtained by heart bioassay. The brain bioassay of the six positive birds showed that only two brains were positive. *T. gondii* was also isolated from the heart of these two birds. The isolates were designated TgWildBrMG 1 to TgWildBrMG 6 (in the isolate designation TgWildBrMG; Tg stands for *T. gondii*, Wild stands for wild animals, Br stands for Brazil, and MG stands for state Minas Gerais, with the isolates numbered according to the chronological order in which isolation was performed).

Isolates TgWildBrMG 1, 3, 4, and 5 were obtained, respectively, from a roadside hawk (*Rupornis magnirostris*), a campo flicker (*Colaptes campestris*), a southern caracara (*Caracara plancus*), and a tropical screech-owl (*Megascops choliba*), all rescued in Belo Horizonte. Isolate TgWildBrMG 2 was obtained from a toco toucan (*Ramphastos toco*), rescued in Cristiano Otoni. Isolate TgWildBrMG 6 was obtained from a southern caracara, rescued in Santa Luzia.

Five different genotypes were identified in the isolates of *T. gondii*: ToxoDB #108 (TgWildBrMG 1), ToxoDB #11, also known as BrII (TgWildBrMG 3), ToxoDB #8, also known as BrIII (TgWildBrMG 5), ToxoDB #13 (TgWildBrMG 6). A unique genotype, not previously described in any other host and/or anywhere else, was common to isolates TgWildBrMG 2 and TgWildBrMG 4 (Table 2).

**Table 2:** PCR-RFLP genotyping of new isolates of *Toxoplasma gondii* obtained from free-living wild birds rescued in the state of Minas Gerais, Brazil.

Isolates	Species of the Bird that *T. gondii* was isolated	Origin	Genetics markers												PCR-RFLP genotype	Identity with other isolates
			SAG 1	5‘+3′ SAG2	alt. SAG2	SAG 3	CS3	BTUB	GRA6	c22-8	c29-2	L358	PK1	Apico		
RH^1^		NA^b^	I	I	I	I	I	I	I	I	I	I	I	I	10	Reference
PTG^1^		NA	II or III	II	II	II	II	II	II	II	II	II	II	II	1	Reference
VEG^1^		NA	II or III	III	III	III	III	III	III	III	III	III	III	III	2	Reference
TgWildBrMG 1	*Rupornis magnirostris*	Belo Horizonte	I	I	II	III	III	III	III	II	I	I	III	I	108^a^	TgCatBr57 and D2
TgWildBrMG 3	*Colaptes campestris*	Belo Horizonte	I	I	II	III	I	III	III	I	III	I	II	III	11 (BrII)^b^	TgCTBr2, 8, 9, 11, 14, 20 and 27, TgCkBr1, 57, 64 and 97, TgDgBr1 and 13, TgRabbitBr2, TgNlTX1, TgCpBr11, 23 and 24, TgCatBr1, 7, 39, 51, 52, 56, 61, 68, 77 and 78
TgWildBrMG 5	*Megascops choliba*	Belo Horizonte	I	III	III	III	III	III	III	II	III	III	III	III	8 (BrIII)^c^	TgCTBr5, TgPgBrPI 2, TgCatBr3, 4, 58–60, 73, 74, TgCkBr7, 11, 17, 131, 132, 133, 134, 194, 195, CH12, TgDgBr4, 12, D8, TgShBr15, TgCpBr17, 18, 20 and 36, TgCkVe1, TgCatUs7, TgOpGa1, TgPgUs15-a.k.a.P45, 46, 55, 57, 58, 76, 89 and 92
TgWildBrMG 6	*Caracara plancus*	Santa Luzia	I	I	I	I	III	I	III	II	III	III	I	III	13^d^	TgPgBrPI7, TgGtBrPI2, TgCkBr165, 167, 170, 174, 176, 179, 180, 183, 184, 185, TgPgBrRN4, TgGtBr10, TgRhHmBr1, TgCkGr25 and 26
TgWildBrMG 2	*Ramphastos toco*	Cristiano Otoni	I	I	II	III	II	III	III	II	III	I	III	I	New	This study
TgWildBrMG 4	*Caracara plancus*	Belo Horizonte	I	I	II	III	II	III	III	II	III	I	III	I	New	This study

In the isolate designation TgWildBrMG, Tg stands for *T. gondii*, Wild stands for wild animals, Br stands for Brazil, and MG stands for Minas Gerais and the isolates were numbered according to the chronological order in which isolation was performed.

^1^Clonal genotypes I (RH), II (PTG), and III (VEG).

^2^NA – Not applicable. Reference strains.

a Isolate from a cat (São Paulo) and a dog (Minas Gerais). Genotyped by Pena et al. (2008) and Silva et al. (2014).

b Isolates from newborns (Minas Gerais), pig (Piauí), chickens (Rondônia, Rio de Janeiro and Paraná), dogs (São Paulo), rabbit (Minas Gerais), capybaras (São Paulo), cats (São Paulo and Paraná), and *Neotoma micropus* (EUA). Genotyped by Dubey et al. (2007, 2008, 2011), Pena et al. (2008), Yai et al. (2009), Carneiro et al. (2013), and Rêgo et al. (2017).

c Isolates from newborns (Minas Gerais), cats (São Paulo and Paraná), chickens (São Paulo, Rondônia, Mato Grasso do Sul, Minas Gerais, and Venezuela), dogs (São Paulo and Minas Gerais), sheep (São Paulo), capybaras (São Paulo), wild cat (EUA), Virginia possum (EUA), and pigs (Estados Unidos). Genotyped by Dubey et al. (2007, 2008, 2011), Pena et al. (2008), Yai et al. (2009), Velmurugan et al. (2009), Ragozo et al. (2010), Soares et al. (2011), Rajendran et al. (2012), Carneiro et al. (2013), and Silva et al. (2014).

d Isolates from chickens (Grenada, Pernambuco, Rio Grande do Norte, Bahia, Ceará, Sergipe, and Alagoas), goats and pigs (Piauí e Rio Grande do Norte), and *Alouatta belzebul*. Genotyped by Dubey et al. (2006, 2008), Ragozo et al. (2010), Pena et al. (2011), Andrade et al. (2013), and Rêgo et al. (2017).

All 6 isolates were non-lethal to mice. However, some mice infected with high doses of tachyzoites obtained from isolates TgWildBrMG 2, 3, and 4 died, whereas all mice infected with isolates TgWildBrMG 1, 5, and 6 survived (Table 3). Analysis of polymorphisms of ROP18/ROP5 genes showed three different combined alleles, including 4/3, 3/3, and 3/1. Isolates TgWildBrMG 1, 2, 3, and 4 have 4/3 allele combination, TgWildBrMG 5 has 3/3, and TgWildBrMG 6 has 3/1 (Table 3).

**Table 3:** PCR-RFLP genotyping of rhoptry gene ROP5, ROP16, ROP17, ROP18, granule dense gene GRA15, and virulence in mice of *Toxoplasma gondii* isolates obtained from free-living wild birds rescued in the state of Minas Gerais, Brazil.

Isolated ID	Virulence proteins					dead/infected^e^ mice with *T. gondii* by dose				Virulence	PCR-RFLP genotype
	GRA15	ROP5	ROP16	ROP17	ROP18	1 Tach^f^	10 Tach	100 Tach	1000 Tach		
RH^a^	1 or 3	1	1	1	1	1/1	4/4	5/5	4/4	Virulent	10
ME49^b^	2	2	2	2	2	0/0	0/3	0/4	0/4	Avirulent	1
VEG^c^	1 or 3	3	1	2	3	ND	ND	ND	ND	ND	2
MAS^d^	ND	4	1	4	4	ND	ND	ND	ND	ND	17
TgWildBrMG 1	1 or 3	3	1	4	4	0/0	0/1	0/5	0/5	Avirulent	108
TgWildBrMG 2	1 or 3	3	1	4	4	0/0	2/5	5/5	3/5	Intermediate	New
TgWildBrMG 3	1 or 3	3	2	4	4	0/1	0/1	1/5	5/5	Intermediate	11 (BrII)
TgWildBrMG 4	1 or 3	3	1	4	4	0/1	3/4	5/5	5/5	Intermediate	New
TgWildBrMG 5	1 or 3	3	1	1	3	0/0	0/5	0/5	0/5	Avirulent	8 (BrIII)
TgWildBrMG 6	1 or 3	1	1	1	3	0/0	0/4	0/5	0/5	Avirulent	13

ND = Not done.

In the isolate designation TgWildBrMG, Tg stands for *T. gondii*, Wild stands for wild animals, Br stands for Brazil, and MG stands for Minas Gerais and the isolates were numbered according to the chronological order in which isolation was performed.

a Clonal genotype type I and virulent control used in this study (RH).

b Clonal genotype type II and avirulent control used in this study (ME49).

c Clonal genotype type III (VEG).

d Atypical genotypes (MAS) with unusual alleles (u-1).

e Infection of the surviving mice was confirmed by ELISA.

f Tach. = Tachyzoites.

## 4 Discussion

Increasing urbanization leads to invasion of the natural habitat of wild animals, increasing the likelihood of transmission of *T. gondii* strains. Furthermore, deforestation may increase the spillover between anthropogenic and sylvatic cycles of *T. gondii* in Brazil due to invasion of the natural habitat of wild animals. Although prohibited in Brazil, hunting and slaughtering wild animals for consumption are still common practices in rural areas (Sogorb et al., 1977; Vitaliano et al., 2014). Such practices can make it easier to share different strains of *T. gondii* between humans and wild animals, and, depending on the virulence of the isolate, the infection may cause the death of immunocompetent individuals (Demar et al., 2007).

This is the first study that aimed to isolate *T. gondii* from the heart and brain of free-living wild birds rescued in the state of Minas Gerais. As far as we know, the isolates of *T. gondii* obtained in this study had not been previously found in any of these five wild bird species in Brazil (*Rupornis magnirostris*, *Colaptes campestris*, *Caracara plancus*, *Megascops choliba*, and *Ramphastos toco*). Other isolation and genetic characterization studies of *T. gondii* obtained from free-living and/or captivity-bred wild animals have been carried out in Brazil, mostly with mammals (Schmidt et al., 1969; Sogorb et al., 1977; Yai et al., 2009; Araújo et al., 2010; Dubey et al., 2011; Pena et al., 2011; Cabral et al., 2013; Barros et al., 2014; Vitaliano et al., 2014; Melo et al., 2016). Thirteen isolates of *T. gondii* have been obtained from wild birds: 12 isolates from *Zenaida auriculata* in the state of Paraná (Barros et al., 2014) and one isolate from a lineated woodpecker (*Dryocopus lineatus*) in the state of São Paulo (Vitaliano et al., 2014).

The role wild birds play in the transmission of *T. gondii* has not yet been totally understood (Lindsay et al., 1991). Birds are intermediary hosts of *T. gondii* and are epidemiologically important as prey and infection routes for Felidae, which is responsible for environmental contamination with oocysts after ingestion of infected tissues (Gennari et al., 2014). In this study, *T. gondii* was isolated from a campo flicker (*Colaptes campestris*). Since campo flickers ingest insects directly in the soil or tree stems, the main source of infection was probably through the ingestion of oocyst-contaminated feed. Gennari et al. (2014) verified high seropositivity for *T. gondii* in birds living in forest soil and in omnivorous birds.

*Toxoplasma gondii* was obtained from the toco toucan in this study. Dubey et al. (2009) isolated *T. gondii* (TgRsCr1 strain) from the chest muscle of a keel-billed toucan (*Ramphastos sulfuratus*), originating from Costa Rica and belonging to another species of this same genus. Minervino et al. (2010) carried out a serological study and did not detect IgG anti-*T. gondii* in the serum of captivity-bred toco toucans. Because it is an omnivorous species, toco toucans may be infected by other animals previously infected with *T. gondii* or they may be infected through food, mainly oocyst-contaminated fruit. Analyzing the stomach content of a toco toucan, Ballarini et al. (2013) found only animal residues.

The birds of prey (southern caracara, roadside hawk and tropical screech-owl) naturally infected with *T. gondii* were probably infected through the ingestion of prey chronically infected with *T. gondii*. Thus, it is possible to infer that other wild animal species may also be infected by *T. gondii*, increasing the number of likely *T. gondii* intermediary hosts. The birds of prey are resistant to clinical toxoplasmosis (Lindsay et al., 1991), as verified in crested caracara experimentally infected with *T. gondii*. The birds produced IgG anti-*T. gondii* on the seventh d.p.i., with peak production between 15 and 30 d.p.i., but they became serum-negative in a short period of postinfection time. Despite being positive by immunohistochemical tests for *T. gondii* in the skeletal muscle, heart, liver, brain, kidneys, esophagus, ventriculum, preventriculum and intestine, no clinical or hematological parameter alterations were verified during the experimental period (Vitaliano et al., 2010).

*Toxoplasma gondii* virulence has been defined as its capacity to kill a mouse after an experimental infection (Saraf et al., 2017). It differs significantly among isolates, indicating their pathogenesis and ability to multiply and manipulate host immune response (Blader and Saeij, 2009). Although virulence determination is multifactorial, some genetic markers have been studied. They codify proteins that manipulate host immune response, contributing to the high virulence observed in some strains (Melo et al., 2011). No isolate obtained in this study was 100% lethal for BALB/c mice. There is a predominance of virulent isolates or isolates of intermediary virulence for mice in Brazil (Brandão et al., 2006; Ferreira et al., 2006; Carneiro et al., 2013; Silva et al., 2014). However, Rêgo et al. (2017) verified that 72% (18/25) of the isolates of *T. gondii* obtained from swine and goats from the state of Piaui, Brazil, are avirulent for mice.

Specific ROP5 alleles can reduce the interferon-γ (IFNγ)-induced immunity-related GTPase (IRGs) coating that promotes killing by disrupting the parasitophorous vacuole membrane (PVM) and, consequently, the death of *T. gondii*, even in the absence of ROP18 expression (Niedelman et al., 2012). ROP18 can only inhibit accumulation of the IRGs on the PVM of strains that also express virulent ROP5 alleles (Reese and Boothroyd, 2011; Behnke et al., 2012). Recent studies showed that the combined analysis of alleles ROP18 and ROP5 (ROP18/ROP5) may help determine the virulence of an isolate of *T. gondii* for mice. The evaluation of these proteins as markers of virulence is probably more adequate and will replace the use of mice in future experiments, favoring the classification of the virulence of a new strain within a week. For the 6 isolates genotyped by ROP18/ROP5 in this study, three combinations including 4/3, 3/3 and 3/1 were identified. Virulence testing of all 6 isolates revealed non-lethal phenotypes. The 2 strains belonged to alleles 3/3 and 3/1 are both non-lethal, which is in agreement with previous reports (Shwab et al., 2016). The 4 isolates belonged to 4/3 combination also showed non-lethal phenotype. Previous reports indicated that the 4/3 combination had both lethal and non-lethal strains (Shwab et al., 2016; Rêgo et al., 2017), suggesting variation in this group. Therefore, additional virulence-associated markers are needed to further differentiate *T. gondii* strains belong to the 4/3 group.

Two genotypes (ToxoDB #8, also known as type BrIII, and ToxoDB #11, also known as type BrII) belong to a group of four genotypes, designated Brazilian clonal strains (BrI, BrII, BrIII, and BrIV), which are rather common in Brazil (Dubey et al., 2011; Pena et al., 2008; Silva et al., 2014; Rêgo et al., 2017). These same genotypes were verified in strains TgWildBrMG 3 and 5, isolated from the campo flicker and tropical screech-owl, respectively. Seven of 27 new *T. gondii* strains obtained from humans in the state of Minas Gerais exhibited genotype BrII, while one of the 27 strains exhibited genotype BrIII (Carneiro et al., 2013). In *T. gondii* isolates obtained from animals in the state of Minas Gerais, genotype BrII was verified in a domestic rabbit by Dubey et al. (2011), as well as in chickens and dogs (Silva et al., 2014). Genotype BrIII was verified also in chicken and dog isolates (Silva et al., 2014). In wild animals from other states, genotype BrII was previously found in capybara (Yai et al., 2009), maned wolf (*Chrysocyon brachyurus*), and black howler monkey (*Alouatta caraya*) (Vitaliano et al., 2014). Genotype BrIII was previously found in capybara (Yai et al., 2009). However, this study is the first to report genotypes BrII and BrIII in the campo flicker and tropical screech-owl, respectively. The isolation of strains with identical genotypes in distinct geographic regions suggests the widespread distribution of Brazilian clonal genotypes of *T. gondii* in Minas Gerais, Brazil. It also suggests that there is a common infection route for humans and domestic and wild animals, probably related to oocyst-contaminated environment (Silva et al., 2014).

Previous studies show virulence results for mice using *T. gondii* strains with the same genotypes identified in wild birds in this study. Pena et al. (2008) observed that strains classified as ToxoDB genotype # 8 (as TgWildBrMG 5) were avirulent for mice. Carneiro et al. (2013) observed that seven strains classified as ToxoDB # 11 genotype (as TgWildBrMG 3) showed variable virulence for mice. Thus, there is no clear correlation between the genotypes of the isolates of *T. gondii* obtained from wild birds and virulence for mice.

*Toxoplasma gondii* isolates obtained from wild birds in this study that share the same genotypes previously found infecting different hosts in the state of Minas Gerais provide valuable information on the epidemiology of this zoonosis and can help identify the infection route for humans (Silva et al., 2014). Wild birds are capable of exploring great areas, and the migratory behavior displayed by some birds can facilitate the transport and dissemination of different strains of *T. gondii* to different geographic regions (Barros et al., 2014). Among the birds from which *T. gondii* was isolated, *R. toco* often move considerable distances while feeding (Holbrook, 2011; Ragusa-Netto, 2013). *C. campestris* is found in open or semi-open areas, but despite widespread distribution in South America, it is not considered migratory (Short, 1975). *C. plancus* and *R. magnirostris* are considered raptors irruptive or local migrants (Bildstein, 2006). *M. choliba* does not travel large areas in search of food, they explore on average an area of 51.2–80.8 ha. As a result of the growth of cities and expansion of agriculture, birds of prey (such as the infected birds observed in this study), probably gave lost fragments of their habitat and adopted a behavior of exploitation of the urbanized environment (forests and ecological parks) of large cities, such as Belo Horizonte. Thus, these birds can play a role in the dissemination of *T. gondii* between these two environments. Environment contamination with oocysts can occur after these birds are consumed by nonimmune felines.

This study showed that 13.3% of the wild birds tested (6/45) was positive with *T. gondii*. This rate is high, and it could be due to high infection in the birds of prey. Nevertheless, it is important to further investigate the role of wild birds in the transmission of *T. gondii* in Brazil. Based only on our results, it is not possible to determine if wild birds are sources, vectors, or victims of *T. gondii* and if they are an import contamination source to domestic animals and people or vice versa. Further studies are needed to elucidate these questions.

## 5 Conclusion

Atypical strains of *T. gondii* isolated from free-living wild birds rescued in the state of Minas Gerais, Brazil share the same genotypes as strains infecting humans, domestic animals, and animals slaughtered for human consumption.

## References

[bib1] Andrade M.M., Pinheiro B.V., Cunha M.M., Carneiro A.C.A.V., Andrade Neto V.F., Vitor R.W.A. (2013). New gentotypes of *Toxoplasma gondii* obtained from farm animals in Northeast Brazil. Res. Vet. Sci..

[bib2] Araújo J.B., Silva A.V., Rosa R.C., Mattei R.J., Silva R.C., Richini-Pereira V.B., Langoni H. (2010). Isolation and multilocus genotyping of *Toxoplasma gondii* in seronegative rodents in Brazil. Vet. Parasitol..

[bib3] Ballarini Y., Frizzas M.R., Marini M.Â. (2013). Stomach contents of brazilian non-passerine birds. Rev. Bras. Ornitolo..

[bib4] Barros L.D., Taroda A., Zulpo D.L., Cunha I.A.L., Sammi A.S., Cardim S.T., Miura A.C., Su C., Machado R.Z., Vidotto O., Garcia J.L. (2014). Genetic characterization of *Toxoplasma gondii* Isolates from eared doves (*Zenaida Auriculata*) in Brazil. Rev. Bras. Parasitol. Vet..

[bib6] Behnke M.S., Fentress S.J., Mashayekhi M., Li L.X., Taylor G.A., Sibley L.D. (2012). The polymorphic pseudokinase ROP5 controls virulence in *Toxoplasma gondii* by regulating the active kinase ROP18. PLoS Pathog..

[bib7] Bildstein K.L. (2006). Migrating Raptors of the World.

[bib8] Blader I.J., Saeij J.P. (2009). Communication between *Toxoplasma gondii* and its host: impact on parasite growth, development, immune evasion, and virulence. APMIS.

[bib9] Brandão G.P., Ferreira A.M., Melo M.N., Vitor R.W.A. (2006). Characterization of *Toxoplasma gondii* from domestic animals Minas Gerais, Brazil. Parasite.

[bib10] Brandão G.P., Melo M.N., Gazzinelli R.T., Caetano B.C., Ferreira A.M., Silva L.A., Vitor R.W.A. (2009). Experimental reinfection of Balb/c mice with different recombinant type I/III strains of Toxoplasma gondii: involvement of IFN-Γ and IL-10. Mem. Inst. Oswaldo Cruz.

[bib11] Cabral A.D., Gama A.R., Sodré M.M., Savani E.S.M.M., Galvão-Dias M.A., Jordão L.R., Maeda M.M., Yai L.E.O., Gennari S.M., Pena H.F.J. (2013). First isolation and genotyping of Toxoplasma gondii from bats (Mammalia: chiroptera). Vet. Parasitol..

[bib12] Carneiro A.C.A.V., Andrade G.M., Costa J.G.L., Pinheiro B.V., Vasconcelos-Santos D.V., Ferreira A.M., Su C., Januário J.N., Vitor R.W.A. (2013). Genetic characterization of *Toxoplasma gondii* revealed highly diverse genotypes for isolates from newborns with congenital toxoplasmosis in Southeastern Brazil. J. Clin. Microbiol..

[bib13] Cavalcante A.C.R., Ferreira A.M., Melo M.N., Fux B., Brandão G.P., Vitor R.W.A. (2007). Virulence and molecular characterization of *Toxoplasma gondii* isolated from goats in Ceará, Brazil. Small Rumin. Res..

[bib14] Demar M., Ajzenberg D., Maubon D., Djossou F., Panchoe D., Punwasi W., Valery N., Peneau C., Daigre J.L., Aznar C., Cottrelle B., Terzan L., Dardé M.L., Carme B. (2007). Fatal outbreak of human toxoplasmosis along the maroni river: epidemiological, clinical, and parasitological aspects. Clin. Infect. Dis..

[bib15] Dubey J.P., Gennari S.M., Labruna M.B., Camargo L.M.A., Vianna M.C.B., Marcet P.L., Lehmann T. (2006). Characterization of *Toxoplasma gondii* isolates in free-range chickens from Amazon, Brazil. J. Parasitol..

[bib16] Dubey J.P., Gennari S.M., Sundar N., Vianna M.C.B., Bandini L.M., Yai L.E.O., Kwok O.C.H., Su C. (2007). Diverse and atypical genotypes identified in *Toxoplasma gondii* from dogs in São Paulo, Brazil. J. Parasitol..

[bib17] Dubey J.P., Graham D.H., Blackston C.R., Lehmann T., Gennari S.M., Ragozo A.M.A., Nishi S.M., Shen S.K., Kwok O.C.H., Hill D.E., Thulliez P. (2002). Biological and genetic characterisation of *Toxoplasma gondii* isolates from chickens (*Gallus domesticus*) from São Paulo, Brazil: unexpected findings. Int. J. Parasitol..

[bib18] Dubey J.P., Lago E.G., Gennari S.M., Su C., Jones J.L. (2012). Toxoplasmosis in humans and animals in Brazil: high prevalence, high burden of disease, and epidemiology. Parasitology.

[bib19] Dubey J.P., Passos L.M.F., Rajendran C., Ferreira L.R., Gennari S.M., Su C. (2011). Isolation of viable *Toxoplasma gondii* from feral Guinea fowl (*Numida meleagris*) and domestic rabbits (*Oryctolagus cuniculus*) from Brazil. J. Parasitol..

[bib21] Dubey J.P., Velmurugan G.V., Chockalingam A., Pena H.F.J., Oliveira L.N., Leifer C.A., Gennari S.M., Bahia-Oliveira L.M.G., Su C. (2008). Genetic diversity of *Toxoplasma gondii* isolates from chickens from Brazil. Vet. Parasitol..

[bib22] Dubey J.P., Velmurugan G.V., Morales J.A., Arguedas R., Su C. (2009). Isolation of *Toxoplasma gondii* from the keel-billed toucan (*Ramphastos sulfuratus*) from Costa Rica. J. Parasitol..

[bib23] Dubey J.P., Why K.V., Verma S.K., Choudhary S., Kwok O.C.H., Khan A., Behinke M.S., Sibley L.D., Ferreira L.R., Oliveira S., Weaver M., Stewart R., Su C. (2014). Genotyping *Toxoplasma gondii* from wildlife in Pennsylvania and identification of natural recombinants virulent to mice. Vet. Parasitol..

[bib24] Ferreira A.M., Martins M.S., Vitor R.W.A. (2001). Virulence for Balb/c mice and antigenic diversity of eight *Toxoplasma gondii* strains isolated from animals and humans in Brazil. Parasite.

[bib25] Ferreira A.M., Vitor R.W.A., Gazzinelli R.T., Melo M.N. (2006). Genetic analysis of natural recombinant brazilian *Toxoplasma gondii* strains by multilocus PCR-RFLP. Infect. Genet. Evol..

[bib26] Gennari S.M., Ogrzewalska M., Soares H.S., Saraiva D.G., Pinter A., Labruna M.B., Dubey J.P. (2014). Occurrence of *Toxoplasma gondii* antibodies in birds from the atlantic forest, state of São Paulo, Brazil. Vet. Parasitol..

[bib27] Gilbert R.E., Freeman K., Lago E.G., Bahia-Oliveira L.M.G., Tan H.K., Wallon M., Buffolano W., Stanford M.R., Petersen E. (2008). Ocular sequelae of congenital toxoplasmosis in Brazil compared with Europe. PLoS Neglected Trop. Dis..

[bib28] Howe D.K., Sibley L.D. (1995). *Toxoplasma gondii* comprises of parasite three clonal lineages: correlation with human disease genotype. J. Infect. Dis..

[bib29] Holbrook K.M. (2011). Home range and movement patterns of Toucans: implications for seed dispersal. Biotropica.

[bib30] Lindsay D.S., Dubey J.P., Blagburn B.L. (1991). *Toxoplasma gondii* infections in red-tailed hawks inoculated orally with tissue cysts. J. Parasitol..

[bib31] Macedo M.F.S.B., Macedo C.A.B., Ewald M.P.C., Martins G.F., Zulpo D.L., Cunha I.A.L., Taroda A., Cardim S.T., Su C., Garcia J.L. (2012). Isolation and genotyping of *Toxoplasma gondii* from pregnant dairy cows (*Bos taurus*) slaughtered. Rev. Bras. Parasitol..

[bib32] Melo M.B., Jensen K.D.C., Saeij J.P.J. (2011). *Toxoplasma gondii* effectors are master regulators of the inflammatory response. Trends Parasitol..

[bib33] Melo R.P.B., Almeida J.C., Lima D.C.V., Pedrosa C.M., Magalhães F.J.R., Alcântara A.M., Barros L.D., Vieira R.F.C., Garcia J.L., Mota R.A. (2016). Atypical *Toxoplasma gondii* genotype in feral cats from the Fernando de Noronha Island, northeastern Brazil. Vet. Parasitol..

[bib34] Minervino A.H.H., Soares H.S., Barrêto-Júnior R.A., Neves K.A.L., Pena H.F.J., Ortolani E.L., Dubey J.P., Gennari S.M. (2010). Seroprevalence of *Toxoplasma gondii* antibodies in captive wild mammals and birds in Brazil. J. Zoo Wildl. Med..

[bib35] Murata F., Cerqueira-Cezar C., Kwok O., Tiwari K., Sharma R., Su C., Dubey J.P. (2018). Role of rats (*Rattus norvegicus*) in the epidemiology of *Toxoplasma gondii* infection in Grenada, West Indies. J. Parasitol..

[bib36] Niedelman W., Gold D.A., Rosowski E.E., Sprokholt J.K., Lim D., Arenas A.F., Melo M.B., Spooner E., Yaffe M.B., Saeij J.P.J. (2012). The rhoptry proteins ROP18 and ROP5 mediate *Toxoplasma gondii* evasion of the murine, but not the human, interferon-gamma response. PLoS Pathog..

[bib37] Pena H.F.J., Gennari S.M., Dubey J.P., Su C. (2008). Population structure and mouse-virulence of *Toxoplasma gondii* in Brazil. Int. J. Parasitol..

[bib38] Pena H.F.J., Marvulo M.F.V., Horta M.C., Silva M.A., Silva J.C.R., Siqueira D.B., Lima P.A.C.P., Vitaliano S.N., Gennari S.M. (2011). Isolation and genetic characterisation of *Toxoplasma gondii* from a red-handed howler monkey (*Alouatta belzebul*), a jaguarundi (*Puma yagouaroundi*), and a black-eared opossum (*Didelphis aurita*) from Brazil. Vet. Parasitol..

[bib39] Ragozo A.M.A., Pena H.F.J., Yai L.E.O., Su C., Gennari S.M. (2010). Genetic diversity among *Toxoplasma gondii* isolates of small ruminants from Brazil: novel genotypes revealed. Vet. Parasitol..

[bib40] Ragusa-Netto J. (2013). Frugivory by toco toucan (*Ramphastos toco)* inhabiting a mountain chain in the Brazilian cerrado. Wilson J. Ornithol..

[bib41] Rajendran C., Su C., Dubey J.P. (2012). Molecular genotyping of *Toxoplasma gondii* from Central and South America revealed high diversity within and between populations. Infect. Genet. Evol..

[bib42] Rêgo W.M.F., Costa J.G.L., Baraviera R.C.A., Pinto L.V., Bessa G.L., Lopes R.E.N., Vitor R.W.A. (2017). Association of ROP18 and ROP5 was efficient as a marker of virulence in atypical isolates of *Toxoplasma gondii* obtained from pigs and goats in Piauí, Brazil. Vet. Parasitol..

[bib43] Reese M.L., Boothroyd J.C. (2011). A conserved non-canonical motif in the pseudoactive site of the ROP5 pseudokinase domain mediates its effect on Toxoplasma virulence. J. Biol. Chem..

[bib44] Saraf P., Shwab E.K., Dubey J.P., Su C. (2017). On the determination of *Toxoplasma gondii* virulence in mice. Exp. Parasitol..

[bib45] Schmidt S., Galvao A., Fernandes W., Oliveira R. (1969). Do primeiro encontro do *Toxoplasma gondii* (Nicolle e Manceaux, 1909) em morcegos. Rev. Goiana Med..

[bib46] Scott S., Kranz J.E., Cole J., Lincecum J.M., Thompson K., Kelly N., Bostrom A., Theodoss J., Al-Nakhala B.M., Vieira F.G., Ramasubbu J., Heywood J.A. (2008). Design, power, and interpretation of studies in the standard murine model of als. Amyotroph Lateral Scler..

[bib47] Shwab E.K., Jiang T., Pena H.F.J., Gennari S.M., Dubey J.P., Su C. (2016). The ROP18 and ROP5 gene allele types are highly predictive of virulence in mice across globally distributed strains of *Toxoplasma gondii*. Int. J. Parasitol..

[bib48] Short L.L. (1975). A zoogeographic analysis of the South American chaco avifauna. Bull. Am. Mus. Nat. Hist..

[bib49] Silva L.A., Andrade R.O., Carneiro A.C.A.V., Vitor R.W.A. (2014). Overlapping *Toxoplasma gondii* genotypes circulating in domestic animals and humans in southeastern Brazil. PLoS One.

[bib50] Sogorb F., Jamra L.F., Guimarães E.C. (1977). Toxoplasmose em animais de São Paulo, brasil. Rev. Inst. Medici. Trop. São Paulo..

[bib51] Soares R.M., Silveira L.H., Silva A.V., Ragozo A., Galli S., Lopes E.G., Gennari S.M., Pena H.F.J. (2011). Genotyping of *Toxoplasma gondii* isolates from free range chickens in the Pantanal area of Brazil. Vet. Parasitol..

[bib52] Su C., Shwab E.K., Zhou P., Zhu X.Q., Dubey J.P. (2010). Moving towards an integrated approach to molecular detection and identification of *Toxoplasma gondii*. Parasitology.

[bib53] Velmurugan G.V., Su C., Dubey J.P. (2009). Isolate designation and characterization of *Toxoplasma gondii* isolates from pigs in the United States. J. Parasitol..

[bib54] Vitaliano S.N., Mineo T.W.P., André M.R., Machado R.Z., Mineo J.R., Werther K. (2010). Experimental infection of crested caracara (*Caracara plancus*) with *Toxoplasma gondii* simulating natural Conditions. Vet. Parasitol..

[bib55] Vitaliano S.N., Soares H.S., Minervino A.H.H., Santos A.L.Q., Werther K., Marvulo M.F.V., Siqueira D.B., Pena H.F.J., Soares R.M., Su C., Gennari S.M. (2014). Genetic characterization of *Toxoplasma gondii* from brazilian wildlife revealed abundant new genotypes. Int. J. Parasitol.: Parasites Wildl..

[bib56] Yai L.E.O., Ragozo A.M.A., Aguiar D.M., Damaceno J.T., Oliveira L.N., Dubey J.P., Gennari S.M. (2008). Isolation of *Toxoplasma gondii* from capybaras (*Hydrochaeris hydrochaeris*) from São Paulo state, Brazil. J. Parasitol..

[bib57] Yai L.E.O., Ragozo A.M.A., Soares R.M., Pena H.F.J., Su C., Gennari S.M. (2009). Genetic diversity among capybara (*Hydrochaeris hydrochaeris*) isolates of *Toxoplasma gondii* from Brazil. Vet. Parasitol..

